# Multiple primary melanomas with numerous pink-to-tan papules and polydactylous onychopapillomas

**DOI:** 10.1016/j.jdcr.2026.02.003

**Published:** 2026-02-10

**Authors:** Simon J. Waigand, Ines Bertlich, Kai-P. Linse

**Affiliations:** Department of Dermatology, University Hospital Heidelberg, Heidelberg, Germany

**Keywords:** BAP1, BAP1 tumor predisposition syndrome, BAP1-inactivated melanocytic tumor, dermoscopy, nail abnormalities, onychopapilloma

## Case description

**Fig 1:**
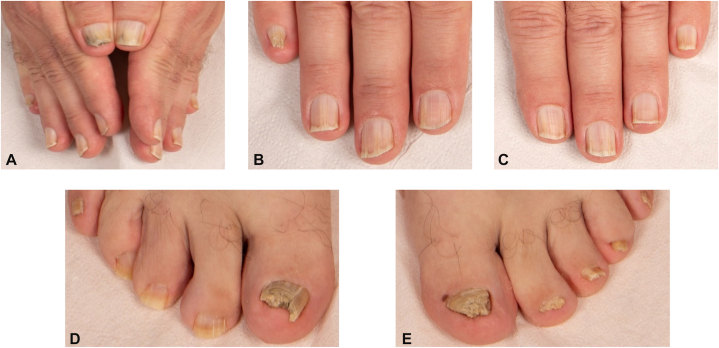
**A,** Clinical nail finding of the hands and feet. **B** and **C,** Overview of both hands showing diffuse nail dystrophy with *yellow-brown* discoloration, erythronychia, splinter haemorrhages, and distal onycholysis, most pronounced in both thumbs. **D** and **E,** Fingernails with single and multiple bands of longitudinal leukonychia and distal hyperkeratotic hyponychial spicules; a pincer deformity is present in the right fifth fingernail. Digitus pedis II, III, and IV of the left foot exhibit marked *yellow-brown* discoloration with diffuse subungual hyperkeratosis, erythronychia, splinter haemorrhages, and distal onycholysis. The great toenails are severely dystrophic with a compact, horn-like hyperkeratotic mass, with additional onychogryphosis of the right great toenail.

**Fig 2:**
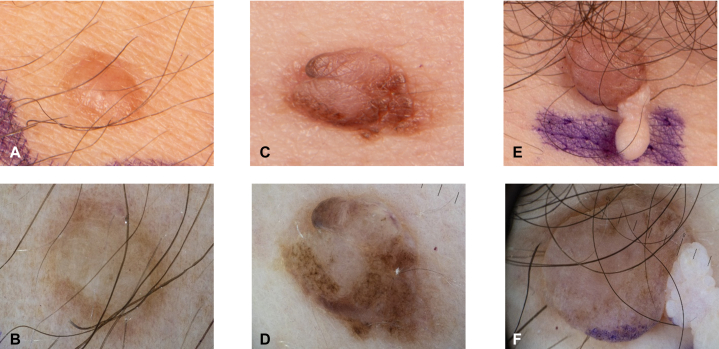
Clinical and dermatoscopic image of histologic conformied BIMTs: **A,** Clinical image of a skin-colored *dome-shaped papule***(B)**: dermatoscopic image shows symmetric lesion with a predominantly structureless skin-colored central lesion and eccentrically located dark clods **(C)**: clinical image of an exophytic, asymmetric, multilobulated, predominantly skin-colored tumor with superimposed eccentric *brown lobules***(D)**: dermatoscopic image shows an asymmetric lesion with an irregular, *dark-brown* peripheral network and blotches surrounding a centrally raised, structureless *light-brown* area **(E)**: clinical image of a *dome-shaped pink tumor* with at the caudal pole small, *flesh-colored*, pedunculated, filiform appendage **(F)**: dermatoscopic image shows a symmetrical, sharply demarcated, *dome-shaped lesion* with a homogeneous *light-brown* to *beige* pigmentation and a confluent globular pattern. Laterally, a *skin-colored*, multilobulated papillomatous appendage with grape-like *whitish papules* is attached; *purple* surgical ink is visible at the inferior margin. *BIMTs*, BAP1-inactivated melanocytic tumors.

**Fig 3:**
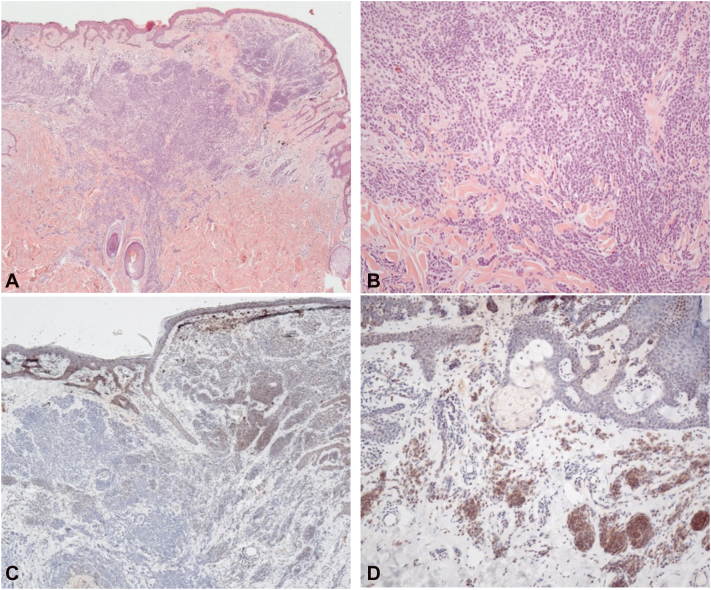
Histopathological and immunohistochemical findings of a BIMT: **A,** H&E, 25× and **(B)** H&E, 100×: Symmetrical intraepidermal and dermal melanocytic lesion with maturation gradient in HMB-45 stain (no image) and without elevated mitotic activity in Ki-67 stain (no image). PRAME stain (no image) is negative. **C,** Immunohistochemistry: BAP1 staining shows complete loss of nuclear expression in intraepidermal and dermal melanocytes, 50×. **D,** BRAF V600E (VE1) immunohistochemistry shows diffuse cytoplasmic positivity in the majority of lesional melanocytes, 100×. *BIMT*, BAP1-inactivated melanocytic tumor; *H&E*, hematoxylin and eosin; *PRAME,* preferentially expressed antigen in melanoma.

A 54-year-old man presented for excision of a melanoma (Breslow thickness 1.6 mm) on the right scapula and sentinel lymph node biopsy. Total-body skin examination identified 3 additional lesions that were excised and confirmed as a conventional melanoma (Breslow thickness 2.4 mm) on the left chest and 2 melanomas in situ on the right scapular region and the left nasal sidewall. All fingernails showed single or multiple bands of longitudinal leukonychia with a focal hyperkeratotic spicule at the hyponychium. The right fifth fingernail showed a pincer deformity. The thumbs and great toenails exhibited diffuse hyperkeratosis, erythronychia, splinter hemorrhages, and distal onycholysis. Several toenails were markedly dystrophic, with onychogryphosis of the right hallux (Fig 1). Mycological investigation of the nails yielded negative results. The trunk harbored numerous (>30) skin-colored papules (Fig 2). Excision demonstrated BAP1-inactivated melanocytic tumors (BIMTs) with complete loss of nuclear BAP1 expression (Fig 3). Family history was notable for cutaneous melanoma in the father, metastatic thyroid carcinoma in the mother, ovarian carcinoma in the grandmother, and an unspecified spinal malignancy in an uncle. Taken together, this constellation raised concern for an underlying hereditary cancer predisposition syndrome, and germline testing identified a heterozygous nonsense BAP1 variant (NM_004656.4:c.1153C>T; p.Arg385∗).

**Question: Which diagnosis best explains the combination of multiple primary melanomas, numerous BAP1-inactivated melanocytic tumors, and polydactylous onychopapillomas?**

**A.** Familial atypical multiple mole melanoma syndrome
**B.** BAP1 tumor predisposition syndrome
**C.** PTEN hamartoma tumor syndrome
**D.** Carney complex
**E.** Muir–Torre syndrome

**Answer: B. BAP1 tumor predisposition syndrome.**

## Discussion

BAP1 tumor predisposition syndrome (BAP1-TPDS) is an autosomal dominant cancer predisposition syndrome caused by germline pathogenic variants in BAP1, which encodes a nuclear deubiquitinase involved in maintaining genomic stability. Pathogenic BAP-1 variants increase the risk of malignancies, most notably cutaneous and uveal melanoma, malignant mesothelioma, and renal cell carcinoma.^1^

Diagnostic challenges arise because BAP1-associated skin cancers, particularly cutaneous melanoma and basal cell carcinoma, are common in the general population, potentially masking hereditary predisposition. Characteristic benign cutaneous findings can provide early diagnostic clues to BAP1-TPDS.

BIMTs, designated as BAP1-inactivated melanocytoma in the 5th edition of World Health Organization classification of skin tumor, represent the most characteristic benign cutaneous lesions in BAP1-TPDS. Clinically, BIMTs may resemble intradermal nevi or fibromas, often presenting as pink-to-tan dome-shaped papules. Dermatoscopically, 2 patterns appear characteristic: a structureless pink-to-tan area with irregular dots/globules and a network with raised, structureless pink-to-tan areas.^2^

Histopathologically, BIMTs show a biphasic pattern with a nodular or sheet-like proliferation of epithelioid melanocytes in the background of an adjacent conventional naevus. The epithelioid cells often have amphophilic cytoplasm with vesicular nuclei and prominent nucleoli and are frequently nonpigmented or lightly pigmented. Although atypia may be marked, dermal mitoses are typically absent or low, but overlap can complicate distinction from atypical Spitz tumors and melanoma. Immunohistochemistry shows complete loss of nuclear BAP1 in the epithelioid cells, with retained nuclear staining in the adjacent nevus. Recent publications suggest that preferentially expressed antigen in melanoma immunohistochemistry and single-nucleotide polymorphism microarray–based copy-number profiling may provide supportive ancillary information in challenging cases.^3^^,^^4^

In addition, nail abnormalities are increasingly recognized as a distinctive feature of BAP1-TPDS. A particularly suggestive finding is polydactylous onychopapillomas. Onychopapilloma is typical presenting with subungual hyperkeratosis, longitudinal leukonychia with a distal keratotic spicule at the hyponychium, often accompanied by distal onycholysis and splinter hemorrhages. Since onychopapilloma is an uncommon and usually solitary benign tumor in the general population, multidigit involvement should prompt consideration of a syndromic association.^5^

This case highlights that characteristic clinical and dermoscopic features of BIMTs together with polydactylous onychopapillomas are highly suggestive of BAP1-TPDS and should prompt germline testing, emphasizing the key role of dermatologists in identifying at-risk individuals and enabling timely multidisciplinary care and surveillance.

## Conflicts of interest

None disclosed.
